# Ripk1 is critical for preserving effector regulatory T cells and the suppressive transcriptional program in regulatory T cells

**DOI:** 10.1038/s41418-025-01550-3

**Published:** 2025-07-22

**Authors:** Carlos Plaza-Sirvent, Hannah Sturm, Maximilian K. Nocke, Fatemeh Ghorbani, Clara Bessen, Marina C. Greweling-Pils, Stefan Floess, Jana Niemz, Jelle Huysentruyt, Peter Tougaard, Jochen Huehn, Robert Geffers, Daniel Todt, Peter Vandenabeele, Ingo Schmitz

**Affiliations:** 1 Department of Molecular Immunology, Ruhr-University Bochum, Bochum, Germany; 2 Department of Translational and Computational Infection Research, Ruhr-University Bochum, Bochum, Germany; 3 Department of Molecular and Medical Virology, Ruhr-University Bochum, Bochum, Germany; 4 European Virus Bioinformatics Center (EVBC), Jena, Germany; 5 Core Facility of Comparative Medicine, Helmholtz Centre for Infection Research, Braunschweig, Germany; 6 Experimental Immunology, Helmholtz Centre for Infection Research, Braunschweig, Germany; 7 Cell death and Inflammation Unit, VIB-UGent Center for Inflammation Research, Ghent, Belgium; 8 Department of Biomedical Molecular Biology, Ghent University, Ghent, Belgium; 9 Genome Analytics, Helmholtz Centre for Infection Research, Braunschweig, Germany

**Keywords:** Cell death and immune response, Immune cell death, Inflammation, Signal transduction, Immunogenetics

## Abstract

Ripk1 plays an important role as a regulator of programmed cell death processes such as apoptosis and necroptosis and is involved in initiating pro-inflammatory NF-κB signaling. Immune tolerance depends on the proper function and homeostasis of regulatory T (Treg) cells. Here, we show that specific ablation of Ripk1 in Treg cells leads to systemically reduced Treg cell numbers resulting in spontaneous whole-body pathology. Using chimeric mice that allowed us to study Treg cells in the absence of inflammatory conditions, we observed a competitive disadvantage in vivo of Ripk1-deficient compared to Ripk1-proficient Treg cells. Furthermore, single-cell RNA sequencing revealed that Ripk1 is required for the maintenance of the effector Treg cell transcriptional signature, which is essential to prevent immune dysregulation. To overcome the limitation of low cell numbers in the chimeric mice, we isolated Treg cells from mice, in which Ripk1 could be deleted in a tamoxifen-inducible manner. Despite the strong reduction detected in Ripk1-deficient Treg cells of the chimeric mice, we did not observe impaired viability by the sole absence of Ripk1 in Treg cells from the inducible system. Of note, we observed reduced viability of activated Ripk1-deficient Treg cells in the presence of TNF. Together, these findings highlight the fundamental role of Ripk1 in maintaining immune homeostasis by preserving the highly suppressive effector Treg cells.

## Introduction

Regulatory T (Treg) cells are essential to maintain immune homeostasis, dampening inflammatory responses, and preventing autoimmunity. These cells constitute a subgroup of CD4^+^ T cells that are characterized by the expression of the transcription factor Forkhead-Box-Protein P3 (Foxp3) [1, 2]. This transcription factor plays a fundamental role in the development and function of Treg cells, as demonstrated by Foxp3 loss-of-function mutations, which lead to the onset of systemic autoimmunity in humans and mice [3–5]. As a transcriptional regulator, Foxp3 suppresses numerous genes associated with T-cell activation and function [6]. A considerable fraction of Treg cells develops in the thymus before being exported to the periphery. Once in the periphery, Treg cells undergo further differentiation and acquire enhanced suppressive capacity. These cells, known as effector Treg (eTreg) cells, have high expression of Treg cell signature genes [7, 8]. For instance, the co-inhibitory molecule TIGIT, which induces the expression of fibrinogen-like protein 2 (Fgl2) and promotes suppression of pro-inflammatory Th1 and Th17 responses, is highly expressed in eTreg cells [9]. In addition to the TIGIT-Fgl2 axis, other molecules related to Treg cell suppressive function, such as IL-10, PD-1, ICOS, Lag-3, CD73, CD103 and CTLA-4 are highly expressed in the eTreg subset [8, 10, 11].

Homeostatic processes that keep Treg cell numbers in a steady-state are essential to maintain immune tolerance in the organism. In that regard, developing Treg cells depend on γc-cytokine survival signals to prevent the activation of a Foxp3-induced apoptotic program [12]. For example, IL-2-regulated expression of the anti-apoptotic protein Mcl-1 is fundamental to ensure Treg cell homeostasis, protecting Treg cells from Bim-initiated intrinsic apoptosis [13]. Furthermore, cellular FLICE-like inhibitory proteins (c-FLIPs), which inhibit caspase-8-mediated extrinsic apoptosis, are critical for preserving Treg cell homeostasis and preventing the onset of autoimmunity in mice [14]. Next to triggering extrinsic apoptosis, the ligand-induced oligomerization of death receptors can initiate additional signaling pathways. Here, receptor-interacting serine/threonine-protein kinase 1 (RIPK1) is a central regulator of these signaling pathways and cell fate decisions. For instance, tumor necrosis factor (TNF)-induced trimerization of TNF receptor 1 (TNFR1) promotes the recruitment of RIPK1 in a complex that may lead to the activation of the nuclear factor-κB (NF-κB) and the MAPK pathways [15–18]. Alternatively, the release of the RIPK1-containing complex from the membrane receptor can initiate cell death programs such as apoptosis or necroptosis [19–24].

Alterations in the immune system have been connected to *RIPK1* biallelic mutations [25]. Therefore, due to the importance of Treg cells in maintaining immune homeostasis, it is critical to understand how RIPK1 deficiency affects Treg cells. It has been reported that the scaffold function of Ripk1 is necessary for the survival of mature T cells, preventing apoptosis and maintaining the proliferative capacity of these cells [26]. Since conventional T (Tcon) cells and Treg cells manifest different functions, we used Treg cell-specific Ripk1-deficient mice as well as an in vitro inducible deletion system to investigate the role of Ripk1 in Treg cells unambiguously. Additionally, to study the effect of Ripk1-deficient Treg cells in a non-inflammatory environment, we used chimeric mice and found that the Ripk1-deficient Treg cells showed a substantial competitive disadvantage. Moreover, here we describe for the first time the transcriptome alterations in Ripk1-deficient Treg cells in a model that prevents the effects of extrinsic inflammatory cues.

## Results

### Ripk1 deficiency in Treg cells induces systemic inflammation in mice

**Fig. 1:**
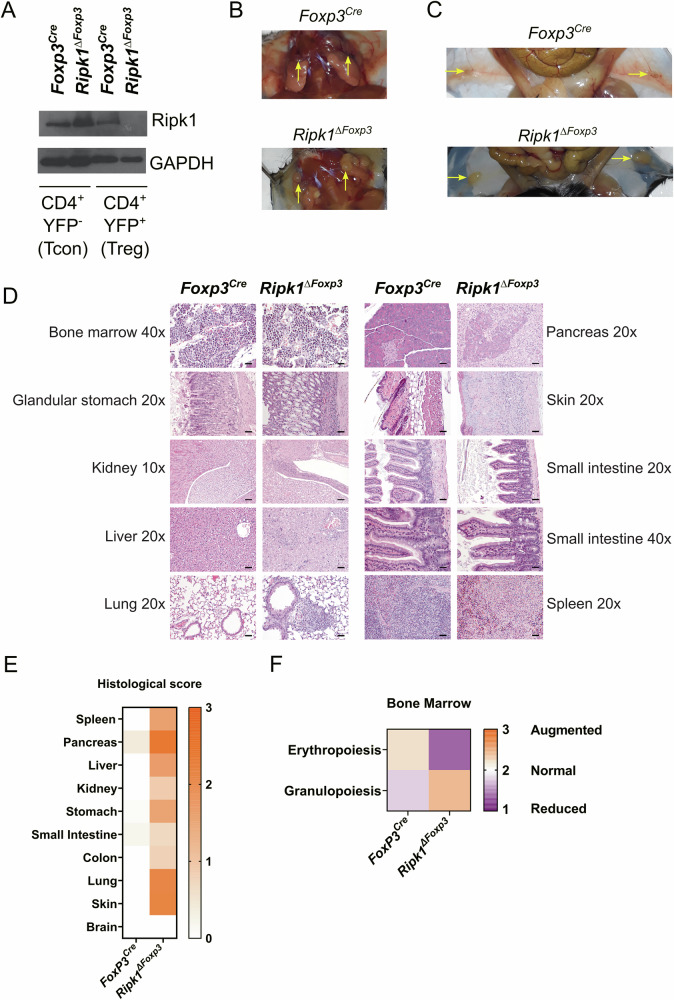
Mice bearing Ripk1-deficient Treg cells develop spontaneous systemic inflammation. **A** Immunoblot analysis of Ripk1 in FACS-sorted Tcon (CD4^+^ YFP^–^) and Treg (CD4^+^ YFP^+^) cells from *Ripk1*^Δ*Foxp3*^ and *Foxp3*^*Cre*^ (control) mice. Pictures of cervical (**B**) and inguinal (**C**) area of 9-week-old *Ripk1*^Δ*Foxp3*^ and *Foxp3*^*Cre*^ mice. Arrows indicate lymph nodes. **D** Representative histological sections of paraffin-embedded tissue of the indicated organs of *Ripk1*^Δ*Foxp3*^ and *Foxp3*^*Cre*^ mice stained with hematoxylin and eosin. **E** Heat map of histological score of the indicated organs of *Ripk1*^Δ*Foxp3*^ and *Foxp3*^*Cre*^ mice (*n* = 5, each group). **F** Heat map of bone marrow erythropoiesis and granulopoiesis of *Ripk1*^Δ*Foxp3*^ and *Foxp3*^*Cre*^ mice (*n* = 5, each group).

To investigate the physiological function of Ripk1 in Treg cells, we crossed *Ripk1*^*fl/fl*^ mice with *Foxp3*^*Cre*^ mice resulting in the specific deletion of Ripk1 in all Foxp3^+^ cells (hereafter referred to as *Ripk1*^Δ*Foxp3*^ mice) (Fig. S1A). We performed immunoblot analysis of sorted T cells to confirm the gene deletion. As expected, Ripk1 was absent in Treg cells isolated from *Ripk1*^Δ*Foxp3*^ mice but present in Tcon cells from the same mice as well as in Tcon and Treg cells isolated from *Foxp3*^*Cre*^ control mice (Fig. 1A). When the *Ripk1*^Δ*Foxp3*^ mice reached the age of approximately seven weeks, we observed stunted growth compared to the littermate controls (Fig. S1B). At the organ level, we detected enlarged lymph nodes, splenomegaly, and atrophied thymus in *Ripk1*^Δ*Foxp3*^ mice (Fig. 1B-C and S1C), suggesting hyperactivation of the immune system. In line with these observations, the histological analysis revealed immune cell infiltration and disruption of the tissue structure in all examined organs except the brain (Fig. 1D-E). Furthermore, we detected reduced erythropoiesis and augmented granulopoiesis in the bone marrow of *Ripk1*^Δ*Foxp3*^ mice (Fig. 1F). In most cases, this phenotype emerged 40-50 days after birth, and the animals were humanely euthanized (Fig. S1D). These findings demonstrate that Ripk1 in Treg cells is essential to maintain immune homeostasis and prevent the onset of spontaneous fatal autoimmunity.

### Reduced Treg cell frequencies and T cell activation in *Ripk1*^Δ*Foxp3*^ mice

**Fig. 2:**
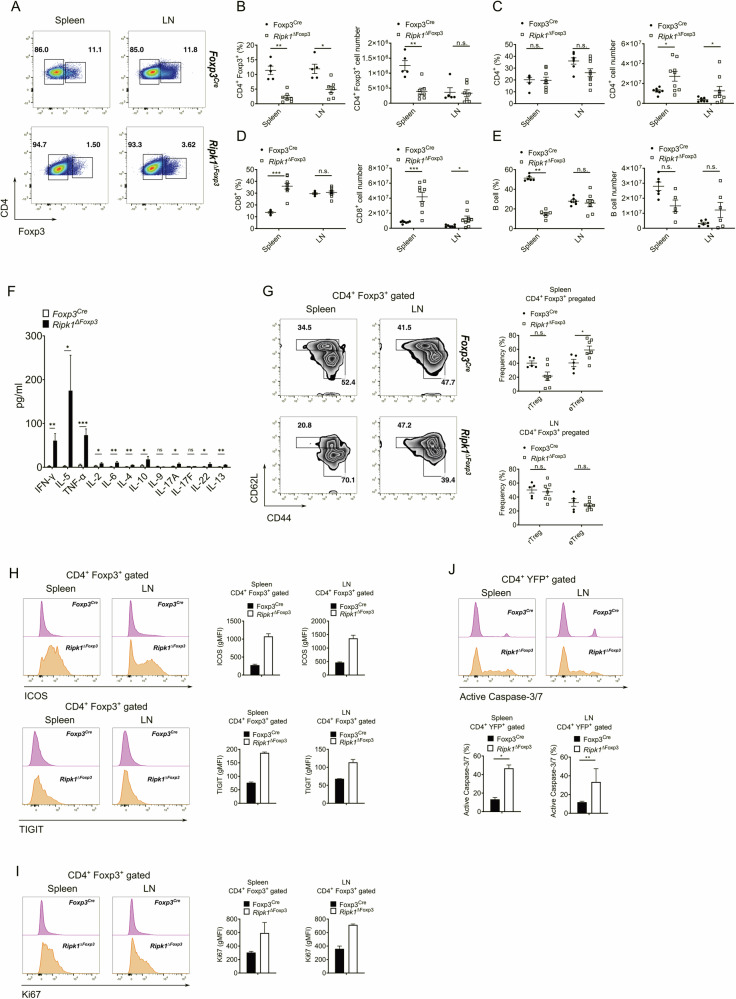
*Ripk1*^Δ*Foxp3*^ mice display reduced Treg frequencies and T cell hyperactivation. **A** Representative dot plots of Foxp3-expressing cells within CD4^+^ cells of *Ripk1*^Δ*Foxp3*^ and *Foxp3*^*Cre*^ mice. Percentages (left) and absolute cell number (right) of CD4^+^ Foxp3^+^ cells (**B**), CD4^+^ cells (**C**), CD8^+^ cells (**D**), and B cells (**E**) of spleen and lymph nodes (LN) from *Ripk1*^Δ*Foxp3*^ and *Foxp3*^*Cre*^ mice. **F** Concentration of the indicated cytokines in sera from *Ripk1*^Δ*Foxp3*^ and *Foxp3*^*Cre*^ mice determined by Luminex assay (*Ripk1*^Δ*Foxp3*^ *n* = 13*, Foxp3*^*Cre*^ *n* = 5). Bar graphs represent the mean ± SEM. Statistical analyses were performed by two-tailed Mann–Whitney tests. *p < 0.05, **p < 0.01, ***p < 0.001, n.s. not significant. **G** Representative zebra plots (left) and frequencies (right) of Treg cell activation markers CD62L and CD44 in CD4^+^ Foxp3^+^ cells from spleen and lymph nodes (LN) of *Ripk1*^Δ*Foxp3*^ and *Foxp3*^*Cre*^ mice. **B**–**E**, **G** Mean ± SEM are given. Each symbol represents a single mouse in the scatter plots. **H** Representative histograms and geometric mean fluorescence intensity (gMFI) bar graphs of ICOS (upper) and TIGIT (lower) expression in CD4^+^ Foxp3^+^ cells from spleen and lymph nodes (LN) of *Ripk1*^Δ*Foxp3*^ and *Foxp3*^*Cre*^ mice (*n* = 2, each). **I** Representative histograms and gMFI bar graphs of Ki67 expression in CD4^+^ Foxp3^+^ cells from spleen and lymph nodes (LN) of *Ripk1*^Δ*Foxp3*^ and *Foxp3*^*Cre*^ mice (*n* = 2, each). **J** Representative histograms (top) and summary bar graph (bottom) of active caspase-3/7 in CD4^+^ YFP^+^ cells from spleen and lymph nodes (LN) of *Ripk1*^Δ*Foxp3*^ and *Foxp3*^*Cre*^ mice (*n* = 5, each). **B**–**G**, **J** Statistical analyses were performed by two-tailed Mann–Whitney tests. *p < 0.05, **p < 0.01, ***p < 0.001, n.s. not significant.

Because the spontaneous development of autoimmunity in *Ripk1*^Δ*Foxp3*^ mice is most likely due to a defect in Treg cell-mediated immune regulation, we sought to examine the Treg cell compartment in these mice. Treg cell frequencies in *Ripk1*^Δ*Foxp3*^ mice were significantly reduced compared to *Foxp3*^*Cre*^ control mice (Fig. 2A, B). Despite the organ enlargement, the absolute number of Treg cells in the spleen was notably reduced in *Ripk1*^Δ*Foxp3*^ mice (Fig. 2B). Interestingly, while the percentages of CD4^+^ T cells were comparable, the frequencies of CD8^+^ T cells were elevated in the spleen of *Ripk1*^Δ*Foxp3*^ mice. The cellularity of both cell subsets was higher in *Ripk1*^Δ*Foxp3*^ mice (Fig. 2C, D), likely due to the increased organ size. In contrast to CD8^+^ T cells, we found that the frequencies of B cells were strongly reduced in the spleens of *Ripk1*^Δ*Foxp3*^ mice (Fig. 2E). When we examined the composition of other immune cells, we did not observe differences between *Ripk1*^Δ*Foxp3*^ and control mice apart from a reduction of NK cell frequencies in the spleen (Fig. S1E–I). We found higher numbers of neutrophils, macrophages, DCs and eosinophils in the lymph nodes (Fig. S1E–I), probably connected to organ enlargement. In order to better characterize the type of immune response developed as a consequence of the Ripk1 deletion in Treg cells, we measured cytokine concentrations in the serum of *Ripk1*^Δ*Foxp3*^ mice, finding elevated levels of the Th1 cytokines IFNγ and TNF together to notable high levels of IL-5 (Fig. 2F).

Since the *Ripk1*^Δ*Foxp3*^ mice developed signs of systemic inflammation, we analyzed the activation status of the T cells. In line with our previous results, we observed a notable switch from naïve to the effector-memory state in Tcon and CD8^+^ T cells of *Ripk1*^Δ*Foxp3*^ mice (Fig. S1J). Based on the expression of CD62L and CD44, we observed an increase in frequencies of eTreg cells in the spleen of *Ripk1*^Δ*Foxp3*^ mice, most likely as a response to the inflammatory environment (Fig. 2G). Accordingly, ICOS and TIGIT, which are typically expressed by activated Treg cells, were upregulated in these cells of *Ripk1*^Δ*Foxp3*^ mice (Fig. 2H). The upregulation of these activation markers might be a consequence of the general hyper-activation of T cells in *Ripk1*^Δ*Foxp3*^ mice. Because these eTreg cells are apparently unable to control immune homeostasis in *Ripk1*^Δ*Foxp3*^ mice, Ripk1-deficient Treg cells are probably impaired in their suppressive capacity. Despite the reduced levels of Treg cells, the high expression of Ki67 indicates an elevated proliferation rate in Treg cells of *Ripk1*^Δ*Foxp3*^ mice (Fig. 2I). Simultaneously, these cells also showed elevated apoptosis, as indicated by the activation of effector caspases (Fig. 2J). Of note, ICOS upregulation, elevation of Ki67, and increased apoptosis were also observed in Tcon cells (Fig. S1K–M), suggesting that the phenotype acquired by the immune cells was highly influenced by the inflammatory environment. Together, our findings demonstrate that Foxp3-specific deletion of Ripk1 causes a reduction of Treg cells accompanied by a hyper-activation of the immune system.

### In vitro deletion of Ripk1 does neither affect peripheral Treg cell differentiation nor Treg cell viability

**Fig. 3:**
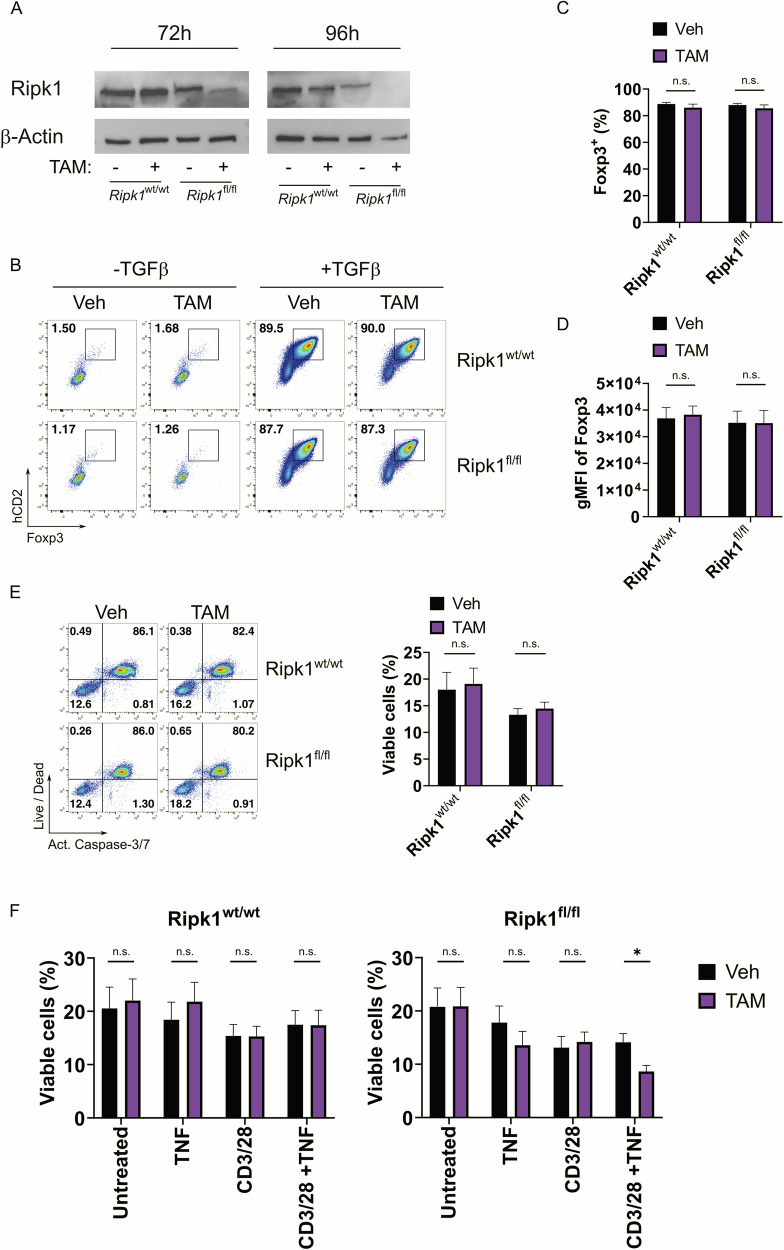
Conserved viability in Treg cells upon in vitro Ripk1 deletion. **A** Immunoblot analysis of Ripk1 in purified Treg cells from *Ripk1*^fl/fl^ Cre-ER^T2^ *Foxp3*^*hCD2*^ and *Ripk1*^wt/wt^ Cre-ER^T2^ *Foxp3*^*hCD2*^ (control) mice treated with tamoxifen or vehicle for the indicated time points. **B** Representative dot plots, **C** Foxp3^+^ hCD2^+^ percentages, and **D** Foxp3 geometric mean fluorescence intensity (gMFI) of events within live CD4^+^ cells of MACS-sorted naïve CD4^+^ T cells from *Ripk1*^fl/fl^ *Cre-ER*^*T2*^ *Foxp3*^*hCD2*^ (*Ripk1*^fl/fl^; *n* = 4) and *Ripk1*^wt/wt^ *Cre-ER*^*T2*^ *Foxp3*^*hCD2*^ (*Ripk1*^wt/wt^ control; *n* = 4) mice treated with tamoxifen for 72 h and subsequently with Treg cell polarizing conditions. Data was obtained from four independent experiments. **E** Representative dot plots (left) and bar graphs (right) of viability analysis determined by CellEvent Caspase-3/7 and LIVE/DEAD Fixable Blue staining of MACS-sorted CD4^+^ hCD2^+^ cells from *Ripk1*^fl/fl^ *Cre-ER*^*T2*^ *Foxp3*^*hCD2*^ (*Ripk1*^fl/fl^; *n* = 5) and *Ripk1*^wt/wt^ *Cre-ER*^*T2*^ *Foxp3*^*hCD2*^ (*Ripk1*^wt/wt^ control; *n* = 5) mice and treated in vitro with Tamoxifen for 72 h. Data was obtained from five independent experiments. **F** Frequencies of viable cells determined by LIVE/DEAD Fixable Blue staining of MACS-sorted CD4^+^ hCD2^+^ cells from *Ripk1*^fl/fl^ Cre-ER^T2^ *Foxp3*^*hCD2*^ (*Ripk1*^fl/fl^; *n* = 8) and *Ripk1*^wt/wt^ Cre-ER^T2^ *Foxp3*^*hCD2*^ (*Ripk1*^wt/wt^ control; *n* = 8) mice and treated in vitro with Tamoxifen for 72 h and, subsequently with TNF (50 ng/ml), anti-CD3 and anti-CD28, combination of anti-CD3, anti-CD28 and TNF (50 ng/ml) or left untreated for 16 additional hours. **C**–**F** Data in bar graphs are represented as mean ± SEM. Statistical analyses were performed by two-tailed Mann–Whitney tests. *p < 0.05, n.s. not significant.

To further investigate the reason for the Treg cell reduction in *Ripk1*^Δ*Foxp3*^ mice, we generated mice containing tamoxifen-inducible Cre alleles together with *Ripk1* floxed alleles and the human CD2 molecule under the control of the *Foxp3* locus as reporter for Foxp3 expression (Fig. S2A). This system allowed us to sort viable Treg cells and induce the deletion of *Ripk1* by tamoxifen treatment in vitro. Immunoblot analysis confirmed that tamoxifen treatment resulted in the loss of Ripk1 expression in lymph node cells and sorted Treg cells from *Ripk1*^*fl/fl*^ *Cre-ER*^*T2*^ *Foxp3*^*hCD2*^ mice after 72 h (Fig. 3A and S2B-C). To test whether Ripk1 plays a role during the generation of Treg cells, we performed in vitro Treg cell differentiation assays after deletion of Ripk1 in naïve Tcon cells from *Ripk1*^fl/fl^ Cre-ER^T2^ *Foxp3*^*hCD2*^ mice (Fig. S2D). The results showed that the percentage of Foxp3^+^ cells and their Foxp3 expression levels were comparable between *Ripk1*^*fl/fl*^ *Cre-ER*^*T2*^ *Foxp3*^*hCD2*^ and control mice, suggesting that Ripk1 is dispensable for the Treg cell differentiation from naïve Tcon cells (Fig. 3B–D). Because high apoptosis was observed ex vivo in Treg cells from *Ripk1*^Δ*Foxp3*^ mice, we sought to investigate the viability of Ripk1-deficient Treg cells in the absence of inflammation. For this purpose, we analyzed membrane integrity and effector caspase activation in sorted tamoxifen-treated Treg cells from *Ripk1*^*fl/fl*^ *Cre-ER*^*T2*^ *Foxp3*^*hCD2*^ mice. We observed that the cell viability of Ripk1-deficient Tregs was not impaired under non-inflammatory conditions (Fig. 3E), suggesting that the absence of Ripk1 alone is not sufficient to trigger apoptotic cell death. Multiple factors can elicit cell death in Treg cells. Next to hyper-activation, Ripk1-deficient Treg cells were exposed to high concentrations of cytokines in the inflammatory environment of the *Ripk1*^Δ*Foxp3*^ mice. To determine the cell death trigger of Ripk1-deficient Treg cells, we stimulated Treg cells isolated from *Ripk1*^*fl/fl*^ *Cre-ER*^*T2*^ *Foxp3*^*hCD2*^ and control mice with anti-CD3/28 antibodies, TNF, or their combination to mimic T cell receptor triggering and the pro-inflammatory environment in *Ripk1*^Δ*Foxp3*^ mice. Our results showed that, next to an overall decrease in cell viability due to cell activation, Ripk1-deficient Treg cells were susceptible to TNF treatment (Fig. 3F). This indicates that the decrease of Treg cells in *Ripk1*^Δ*Foxp3*^ mice may be caused by the pro-apoptotic function of TNF, which was detected in high concentrations in the serum of *Ripk1*^Δ*Foxp3*^ mice.

### Profound reduction of effector Treg cell population in the absence of Ripk1 under non-inflammatory conditions

**Fig. 4:**
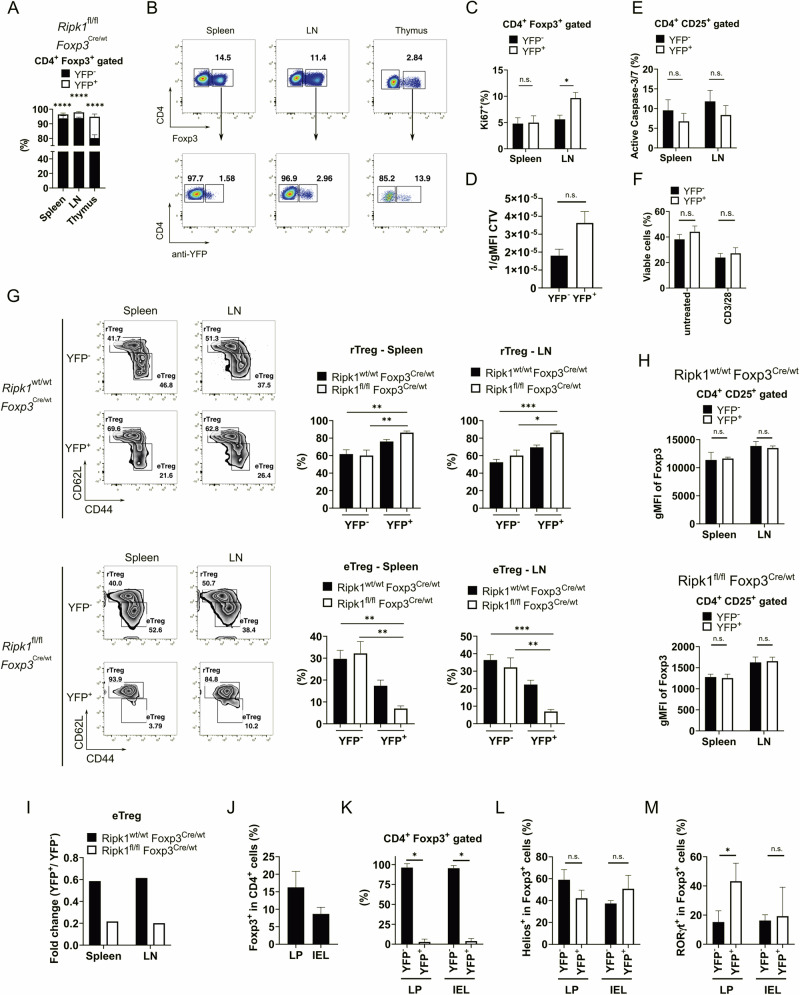
Effector Treg cells are compromised in *Ripk1*^*fl/fl*^*Foxp3*^*Cre/wt*^ mice. **A** Distribution of the percentages of YFP^-^ and YFP^+^ cells within the CD4^+^ Foxp3^+^ population of *Ripk1*^*fl/fl*^ *Foxp3*^*Cre/wt*^ mice (*n* = 11). **B** Representative dot plots of Foxp3-expressing cells within CD4^+^ cells (upper) and YFP^-^expressing cells within Foxp3^+^ cells (lower) from spleen, lymph nodes (LN) and thymus of *Ripk1*^*fl/fl*^ *Foxp3*^*Cre/wt*^ mice. **C** Ki67 positive events in YFP^-^ and YFP^+^ cells within CD4^+^ CD25^+^ cells from spleen and lymph nodes (LN) of *Ripk1*^*fl/fl*^ *Foxp3*^*Cre/wt*^ mice (*n* = 11). **D** Proliferation represented by inverse geometric mean fluorescence intensity (gMFI) of CTV dilution from FACS sorted CD4^+^ CD25^+^ YFP^–^ and CD4^+^ CD25^+^ YFP^+^ cells of *Ripk1*^*fl/fl*^ *Foxp3*^*Cre/wt*^ mice (*n* = 4) stimulated with anti-CD3 (1 µg/ml), anti-CD28 (2 µg/ml) and IL-2 (10 ng/ml) for 72 h. **E** Active caspase-3/7 positive events in YFP^-^ and YFP^+^ cells within CD4^+^ CD25^+^ cells from spleen and lymph nodes (LN) of *Ripk1*^*fl/fl*^ *Foxp3*^*Cre/wt*^ mice (*n* = 11). **F** Viable cell frequency of FACS sorted CD4^+^ CD25^+^ YFP^-^ and CD4^+^ CD25^+^ YFP^+^ cells of *Ripk1*^*fl/fl*^ *Foxp3*^*Cre/wt*^ mice (*n* = 6) stimulated with anti-CD3 (1 µg/ml), anti-CD28 (2 g/ml) and IL-2 (10 ng/ml) for 16 h. **G** Representative zebra plots (left) and distribution in frequencies (right) of resting (rTreg; CD62L^+^ CD44^-^) and effector (eTreg; CD62L^-^ CD44^+^) Treg cells within CD4^+^ Foxp3^+^ cells from spleen and lymph nodes (LN) of *Ripk1*^*wt/wt*^ *Foxp3*^*Cre/wt*^ mice (*n* = 6) and *Ripk1*^*fl/fl*^ *Foxp3*^*Cre/wt*^ mice (*n* = 7). Data in bar graphs are represented as mean ± SEM. Statistical analyses were performed by Kruskal-Wallis test followed by Dunn´s multiple comparison test. *p < 0.05, **p < 0.01, ***p < 0.001, otherwise not significant. **H** Geometric mean fluorescence intensity (gMFI) of Foxp3 in CD4^+^ CD25^+^ cells from spleen and lymph nodes (LN) of *Ripk1*^*fl/fl*^ *Foxp3*^*Cre/wt*^ mice (*n* = 6) and *Ripk1*^*wt/wt*^ *Foxp3*^*Cre/wt*^ mice (*n* = 4). **I** Fold change of average percentages of YFP^+^ vs. YFP^-^ events within eTreg cells from spleen and lymph nodes (LN) of *Ripk1*^*wt/wt*^ *Foxp3*^*Cre/wt*^ mice and *Ripk1*^*fl/fl*^ *Foxp3*^*Cre/wt*^ mice. **J** Frequency of Foxp3^+^ cells in CD4^+^ cells in lamina propria (LP) and in the intraepithelial lymphocyte compartment (IEL) of small intestine of *Ripk1*^*fl/fl*^ *Foxp3*^*Cre/wt*^ mice (*n* = 4). **K** Distribution in frequency of YFP^-^ and YFP^+^ within CD4^+^ Foxp3^+^ cells and their respective Helios (**L**) and RORγt expression (**M**) in lamina propria (LP) and in the intraepithelial lymphocyte (IEL) compartment of small intestine of *Ripk1*^*fl/fl*^ *Foxp3*^*Cre/wt*^ mice (*n* = 4). (**A**, **C**–**F**, **H** and **J**–**M**) Data in bar graphs are represented as mean ± SEM. Statistical analyses were performed by two-tailed Mann–Whitney tests. *p < 0.05, **p < 0.01, ****p < 0.0001, n.s. not significant.

Since *Ripk1*^Δ*Foxp3*^ mice developed systemic inflammation, it remained unclear whether the observed changes in the Treg cells, such as expression of activation markers or increased apoptosis, were a consequence of the absence of Ripk1 or due to the inflammatory environment. To discern the intrinsic effects of the Ripk1 deletion in Treg cells, we generated chimeric mice harboring Ripk1-deficient (Cre-YFP^+^) and Ripk1-proficient (Cre-YFP^−^) Treg cells (Fig. S3A). Because the *Foxp3* locus is located on the X chromosome, Cre-heterozygous female mice are inherent chimeric mice due to random X chromosome inactivation. Therefore, by use of the Cre-YFP reporter, it is possible to fate map the Ripk1-deficient (YFP^+^) and Ripk1-proficient (YFP^−^) Treg cells [27–29]. *Ripk1*^*fl/fl*^ *Foxp3*^*Cre/wt*^ chimeric mice were monitored for a period of 50 weeks, showing no signs of spontaneous phenotype and having an unaltered lifespan (Fig. S3B). Furthermore, chimeric mice displayed no activation of Tcon or CD8^+^ T cells (Fig. S3C) and had normal levels of T follicular helper (Tfh) cells, T follicular regulatory (Tfr) cells, germinal center B cells, plasma cells and Treg cells (Fig. S3D–H), evidencing no immune dysregulation. Importantly, Ripk1-deficient Treg cells were highly reduced compared to Ripk1-expressing Treg cells, as shown by the ratio of YFP^+^ and YFP^−^ cells in this competitive context (Fig. 4A, B). In contrast, mice harboring both Cre-YFP^−^ and Cre-YFP^+^ populations but wild-type Ripk1 alleles did not present such imbalance (Fig. S3I–J). Thus, Ripk1-expressing Treg cells in the chimeric mice appear to compensate for any potential disruption in the immune homeostasis potentially caused by the knock-out cells. Hence, this system allows the study of Ripk1-deficient Treg cells in the absence of inflammation.

Since the levels of YFP^+^ Treg cells were very low in the chimeric mice, we sought to assess proliferation and apoptosis levels in these cells. Interestingly, we could neither detect reduced expression of the proliferation marker Ki67 nor impaired proliferation upon in vitro stimulation (Fig. 4C, D and S3K). Regarding cell death, the activation levels of the effector caspases 3/7 in YFP^−^ and YFP^+^ Treg cells were comparable (Fig. 4E). Furthermore, no decreased viability was found when YFP^−^ and YFP^+^ Treg cells were activated in vitro (Fig. 4F). Altogether, these findings suggest that the reduction of Ripk1-deficient Treg cells is not caused by defects in proliferation or enhanced activation-induced apoptosis (Fig. 4C–F). Comparing the Treg cell subset distribution, we noticed that the eTreg cells were remarkably reduced within the YFP^+^ fraction of the chimeric mice (Fig. 4G). Accordingly, molecules that are crucial for the function of eTreg cells, such as CTLA-4 and ICOS, as well as classical markers of eTreg cells, like TIGIT, Gpr15, CD103 and KLRG1, were downregulated in YFP^+^ Treg cells (Fig. S3L). Undesirable effects using Foxp3^Cre^ mice have been previously reported [30]. In order to check for influence of the YFP/Cre knock-in in Treg cells, we compared the YFP^−^ and YFP^+^ compartments *in Ripk1*^*wt/wt*^ *Foxp3*^*Cre/wt*^ chimeric female mice. Surprisingly, we detected a reduction in the eTreg subpopulation in the YFP^+^ Treg cells (Fig. 4G), even though Foxp3 expression was comparable between the respective YFP fractions (Fig. 4H). While the reduction in the eTreg population of the *Ripk1*^*wt/wt*^ *Foxp3*^*Cre/wt*^ mice was not as drastic as in the *Ripk1*^*fl/fl*^ *Foxp3*^*Cre/wt*^ mice (Fig. 4I), side effects using the Foxp3^Cre^ mouse line need to be considered. Indeed, we found downregulation of Treg markers in the YFP^+^ fraction of Ripk1^wt/wt^ Foxp3^Cre/wt^ (Fig. S3M).

Since eTreg cells are particularly important to maintain tissue homeostasis in non-lymphoid organs, we sought to study the Treg cells in the gut of *Ripk1*^*fl/fl*^ *Foxp3*^*Cre/wt*^ chimeric mice. In our examination, Treg cells were clearly detectable in the lamina propria and the intraepithelial compartment of small intestines of these mice (Fig. 4J). In line with our observations in the lymphoid organs, the Ripk1-deficient (YFP^+^) Treg cells were barely detectable in the intestines of *Ripk1*^*fl/fl*^ *Foxp3*^*Cre/wt*^ mice (Fig. 4K). Interestingly, the decreasing trend of Helios^+^ Treg cells and significant increase of RORγt^+^ Treg cells within the YFP^+^ fraction of the small intestinal lamina propria (Fig. 4L-M) suggest that, to a certain extent, thymic-derived Treg cells are preferentially affected by Ripk1 deletion. In summary, these findings support the idea that, in the absence of inflammation, eTreg cell presence depends on Ripk1.

### Essential effector Treg cell genes are downregulated in Ripk1-deficient Treg cells of Ripk1^fl/fl^ Foxp3^Cre/wt^ mice

**Fig. 5:**
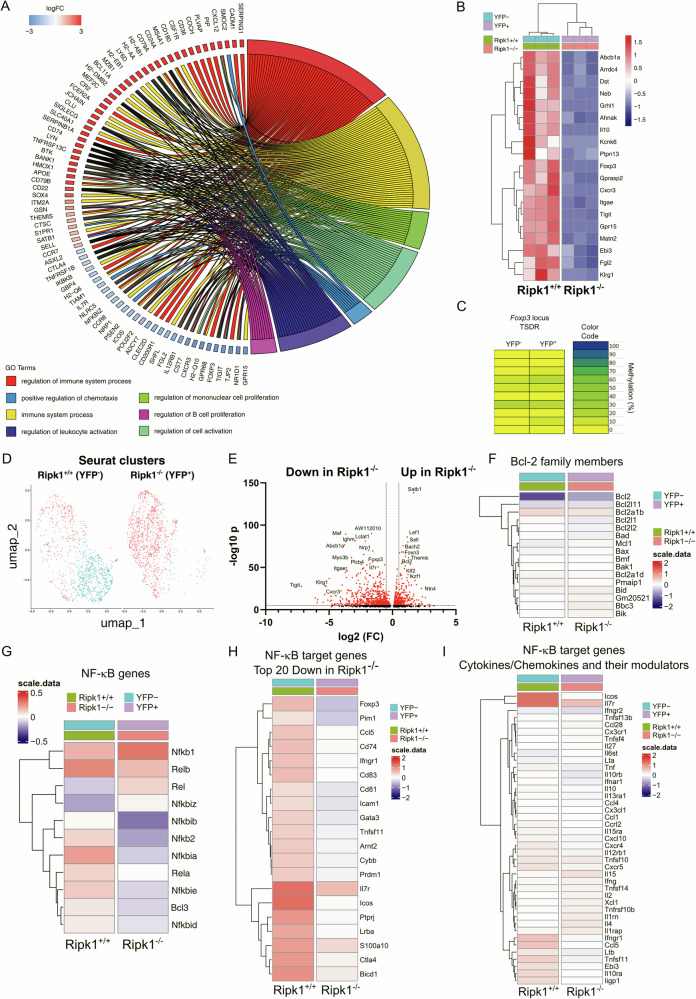
Key Treg cell genes are underrepresented in Ripk1-deficient Treg cells. **A** Chord plot of differentially expressed genes in FACS sorted CD4^+^ CD25^+^ YFP^-^ (Ripk1^+/+^) and CD4^+^ CD25^+^ YFP^+^ (Ripk1 ^–/–^) cells from *Ripk1*^*fl/fl*^ *Foxp3*^*Cre/wt*^ mice. Upregulated genes in CD4^+^ CD25^+^ YFP^+^ (Ripk1 ^–/–^) cells are labeled in red (positive logFC) and downregulated genes are labeled in blue (negative logFC). Chord plot shows a detailed relationship between the log-fold change of differentially expressed genes (DEG) (left semicircle) and their enriched gene ontology (GO) terms (right semicircle). **B** Heat map analysis of Treg gene signature in FACS sorted CD4^+^ CD25^+^ YFP^–^ (Treg Ripk1^+/+^) and CD4^+^ CD25^+^ YFP^+^ (Treg Ripk1^–/–^) cells from *Ripk1*^*fl/fl*^ *Foxp3*^*Cre/wt*^ mice. **C** Representative CNS2/TSDR methylation patterns of FACS sorted CD4^+^ CD25^+^ YFP^–^ and CD4^+^ CD25^+^ YFP^+^ cells from *Ripk1*^*fl/fl*^ *Foxp3*^*Cre/wt*^ mice. Amplicons are vertically arranged, each representing a single CpG-motif. Color code indicates the percentage of CNS2/TSDR methylation in the purified populations (*n* = 4). Data was obtained from two independent experiments. **D** Seurat clustering of scRNA-seq of FACS-sorted CD4^+^ CD25^hi^ cells from spleen and lymph nodes of *Ripk1*^*fl/fl*^ *Foxp3*^*Cre/wt*^ mice. UMAP projections represent clusters of pre-labeled YFP^-^ (left) and YFP^+^ (right) cells. **E** scRNASeq volcano plot showing changes in gene expression of CD4^+^ CD25^hi^ YFP^+^ (Treg Ripk1^–/–^) cells vs. CD4^+^ CD25^hi^ YFP^-^ (Treg Ripk1^+/+^) cells. Heat map analysis of (**F**) Bcl-2 family members, (**G**) NF-κB pathway components, (**H**) Top20 downregulated NF-κB target genes in YFP^+^ cells and (**I**) NF-κB target genes of cytokines/chemokines in FACS sorted CD4^+^ CD25^+^ YFP^-^ (Treg Ripk1^+/+^) and CD4^+^ CD25^+^ YFP^+^ (Treg Ripk1^–/–^) cells from *Ripk1*^*fl/fl*^ *Foxp3*^*Cre/wt*^ mice.

Ripk1 mediates the transcriptional induction of inflammatory cytokines [15, 31, 32], therefore we investigated the impact of Ripk1 deletion on the transcriptome of Treg cells. For this purpose, RNASeq analysis was performed to compare the transcriptome of Ripk1-expressing and Ripk1-deficient Treg cells from *Ripk1*^*fl/fl*^ *Foxp3*^*Cre/wt*^ mice, i.e. in the absence of extrinsic inflammation. In line with our previous results, we detected the downregulation of key genes for Treg cell activation or Treg cell-mediated suppressive function, like *Icos, Tigit*, *Fgl2* or *Ctla4* in Ripk1-deficient Treg cells (Fig. 5A). Notably, the Treg cell master transcription factor *Foxp3* was strongly downregulated in these cells (Fig. 5A). Furthermore, a supervised analysis of the RNASeq data using the Treg gene signature [7] confirmed that a plethora of Treg cell genes were downregulated in Ripk1-deficient Treg cells (Fig. 5B). In the *Foxp3* locus, full demethylation of the conserved non-coding sequence 2 (CNS2), also known as Treg cell-specific demethylated region (TSDR), is required for stable expression of Foxp3 [33]. Thus, we speculated that Ripk1 deficiency affects the epigenetic regulation of the *Foxp3* locus. Surprisingly, our results indicated no differences in the CNS2 methylation status between Ripk1-expressing and Ripk1-deficient Treg cells, suggesting that the reduced *Foxp3* expression in Ripk1-deficient Treg cells is not caused by epigenetic dysregulation of the *Foxp3* locus (Fig. 5C). Nevertheless, the similarity in the methylation pattern proved that the YFP^+^ cells have been mature Treg cells, despite the detected downregulation of *Foxp3* expression. Furthermore, *Sell*, the gene encoding for CD62L, was upregulated in these cells (Fig. 5A), confirming our previous findings showing that the rTreg cell subpopulation was highly predominant in the Ripk1-deficient Treg cell fraction of *Ripk1*^*fl/fl*^ *Foxp3*^*Cre/wt*^ mice (Fig. 4G).

As the bulk RNASeq did not allow us to discriminate cell clusters based on the transcriptional profile of the cells, we next performed single-cell RNA sequencing (scRNASeq) of YFP^−^ and YFP^+^ Treg cells from *Ripk1*^*fl/fl*^ *Foxp3*^*Cre/wt*^ mice. We identified two cell clusters per group, one of them being strongly underrepresented in the YFP^+^ group and resembling the notable loss of eTreg cells in the Ripk1-deficient Treg cell group (Fig. 5D). Interestingly, *Sell* was highly expressed in the larger cluster of the YFP^−^ group (Fig. 5E), indicating that the two identified clusters correspond to the rTreg and eTreg cell subpopulations, which is consistent with the strong reduction of the eTreg population in the Ripk1-deficient Treg cells (Fig. 4G). When YFP^−^ and YFP^+^ cells were compared, we found that Treg cell genes such as *Foxp3*, *Tigit, Itgae, Cxcr3* and *Klrg1* were downregulated in Ripk1-deficient Treg cells (Fig. 5E). Moreover, *Satb1*, a gene that is repressed by Foxp3 in peripheral Treg cells [34], is highly upregulated in Ripk1-deficient Treg cells, which is a further indication of impaired Foxp3 expression (Fig. 5E). Confirming our previous observations of preserved viability, the anti-apoptotic genes *Bcl2* and *Mcl1* were upregulated in Ripk1-deficient Treg cells compared to Ripk1-proficient Treg cells (Fig. 5E, F). Furthermore, we have not observed any indication of enhanced transcription of pro-apoptotic genes in Ripk1-deficient Treg cells (Fig. 5F). Next, we compared the transcriptomic profile of the two identified clusters from each group. Consistent with our previous results, we found *Foxp3* downregulation and *Satb1* upregulation in the cluster of the Ripk1-deficient rTreg cells compared to the Ripk1-expressing rTreg cells (Fig. S4A). In addition, we detected *Foxp3* and *Tigit* downregulation in the Ripk1-deficient eTreg cells (Fig. S4B). Because Ripk1 is involved in NF-κB pathway activation and Treg cell development and function depend on this pathway, we investigated the impact of Ripk1 absence in Treg cells on the NF-κB pathway. First, we observed that the expression of most NF-κB and IκB proteins was downregulated, which may result in impaired signaling through this pathway (Fig. 5G). Next, taking advantage of the published NF-κB target gene signature [35], we identified the downregulation of classical NF-κB target genes and Treg cell genes in Ripk1-deficient Treg cells (Fig. 5H). Finally, we detected impaired expression of key Treg cell cytokines, such as the IL-27/IL-35 subunit *Ebi3*, in Ripk1-deficient Treg cells (Fig. 5I). Of note, effector cytokines like IFNγ, IL-4, IL-15 and IL-2, which are typically repressed by Foxp3 in Treg cells, were upregulated in Ripk1-deficient Treg cells (Fig. 5I). Taken together, these data reveal a profound re-programming of the suppressive transcriptional profile in Ripk1-deficient Treg cells of *Ripk1*^*fl/fl*^ *Foxp3*^*Cre/wt*^ mice and propose a critical role for Ripk1 in preserving the phenotypic stability of eTreg cells.

## Discussion

The study of the multifunctional protein Ripk1 in cells of the immune system has attracted the interest of researchers over the last years. Recently, some of these studies using genetic approaches to delete the *Ripk1* gene in a cell-specific manner focused on investigating Ripk1 in murine T cells. A common denominator in these studies is the impaired survival of the T cells caused by excess apoptosis [26, 36–38]. In those studies, authors used *Cd4* or *Lck* as promoters to induce the expression of the Cre recombinase in order to delete *Ripk1*, targeting all T cells in the conditional KO mice. Using this strategy, there is no discrimination between Tcon and Treg cells when inducing the gene deletion. Thus, in that context, it cannot be established if the cause of the observed T cell phenotype is due to the intrinsic effect of the lack of Ripk1 or if it is produced by an immune dysregulation caused by a suppressive deficiency due to a defect in the Treg cells. The latter scenario was considered in the study of Imanishi et al., which initially used *Cd4* as a promoter to induce Ripk1-specific deletion in T cells [38]. The authors observed T cell senescence, which could be partially restored by inhibiting Caspase-8, a key intermediary in receptor-mediated apoptosis, or by deleting the necroptotic mediator Ripk3. These findings suggested that the T cell senescence and impaired T cell homeostasis might be connected to the activation of apoptotic and necroptotic pathways in the absence of Ripk1. However, because the CD4-specific conditional KO mice developed inflammatory disease, it was not possible to distinguish whether the phenotype occurring in the T cells was caused intrinsically by the absence of Ripk1 or via inflammatory cues. In addition, using in vivo T cell transfers and in vitro T cell co-cultures, the authors concluded that environmental signals determined the senescence phenotype of the T cells [38], suggesting extrinsic effects when Ripk1 was deleted in T cells. The authors also considered the possibility of a defect in the Ripk1-deficient Treg cells as the trigger of the inflammatory environment. In order to validate this hypothesis, they generated Treg cell-specific KO mice crossing *Foxp3*^*Cre*^ mice with *Ripk1*^*fl/fl*^ mice. Contrary to our findings, the Treg-specific conditional KO mice generated in that study did not develop a spontaneous inflammatory phenotype. In line with our observations, a recent publication also reported the rise of spontaneous autoimmunity in mice harboring Ripk1-deficient Treg cells [39]. Accordingly, our study and the work of Deng et al. showed a significant reduction in the peripheral Treg cell levels together with elevated apoptosis in these cells. Unfortunately, the spontaneous autoimmunity induced in *Ripk1*^Δ*Foxp3*^ mice did not allow for clarification of whether the Treg cell phenotype was caused by the deletion of Ripk1 or by environmental cues. Of note, we observed that the Ripk1-proficient Tcon cells of the *Ripk1*^Δ*Foxp3*^ mice also displayed phenotypical changes, such as ICOS upregulation, elevated proliferation, and apoptosis, which appears to be caused by the inflammatory milieu (Fig. S1K-M). Due to the particular capacity of the Treg cells to maintain immune homeostasis, these findings highlight the limitations of generating T cell-specific conditional KO mice using common T cell promoters and the necessity to employ additional systems to investigate the intrinsic role of single proteins in T cells. Moreover, deletion of a gene of interest in the whole Treg cell compartment might cause immune dysregulation. As an alternative, our study took advantage of female chimeric mice to study the impact of the Treg-specific deletion of Ripk1 in the absence of inflammation.

Previous studies have shown that Ripk1 is critical for maintaining T cell homeostasis by preventing apoptosis and that the inhibition of the FADD/Caspase-8 apoptotic pathway restored the T cell levels [26, 38, 39]. Furthermore, some of these works pointed to a partial involvement of the necroptotic pathway in the elimination of the T cells [38, 39]. However, it is not clear whether the apoptotic trigger is coming from the environment or whether it is intrinsically induced by the absence of Ripk1. Besides, genetic ablation of proteins to restore a particular function, for example Caspase-8 deletion to restore cell viability, does not exclude the loss of alternative functions of the deleted protein, like non-apoptotic functions of Caspase-8 [40]. Recently, Huysentruyt et al. demonstrated that Ripk1-deficient naïve T cells and Treg cells are sensitive to TNF-mediated apoptotic cell death [41]. That phenomenon could explain the homeostatic disadvantage of the Ripk1-deficient Treg cell population in the chimeric mice, which would be sensitized to endogenous TNF. However, despite the sensitization of Ripk1-deficient T cells towards extrinsic apoptosis, our results showed that the viability of the Treg cells was not impaired by solely acute induction of Ripk1-deletion in vitro (Fig. 3E, F). Altogether, the findings indicate that, while Ripk1 protects from TNF-induced apoptosis, its absence does not directly initiate cell death in Treg cells.

Post-translational modifications, such as ubiquitination or phosphorylation, control the kinase function of Ripk1, which is essential for the activation of cell death pathways [15]. The absence of a spontaneous phenotype and Treg cell abnormalities in mice containing kinase-dead *Ripk1* alleles suggest that its kinase function is dispensable to maintain the Treg cell-mediated immune homeostasis [26]. In addition to the kinase function, Ripk1 acts as an adaptor protein to activate pro-survival pathways including NF-κB [15]. It is known that the development and function of Treg cells rely on a functional NF-κB pathway [42–46]. For instance, genetic ablation or pharmacological inhibition of the canonical NF-κB signaling initiator IKKβ critically affects Treg cell homeostasis [47]. Moreover, RelA and c-Rel, members of the canonical NF-κB pathway, are essential for Treg cell development or Treg cell suppressive function [43]. In addition, it has been shown that RelA directly coordinates the expression of critical Treg cell genes and is crucial for Treg cell stability [43]. In the same line, other studies showed that RelA deficiency affected Treg cell gene expression and pointed out that RelA is fundamental to maintain the stability of the eTreg cells [11, 48]. Interestingly, in the work of Vasanthakumar et al., the authors showed that RelA activation also occurs in a T cell receptor (TCR)-independent and TNF receptor superfamily (TNFRSF)-dependent manner, associated with Ripk1 signaling [11]. Besides having a highly reduced eTreg compartment, RelA-deficient and Ripk1-deficient Treg cells display high similarities in the transcriptional profile [11, 48]. This, together with the fact that Ripk1 is upregulated in eTreg cells [39], opens the possibility that Ripk1 and RelA are connected and involved in a pathway that leads to eTreg formation.

Regulation of the NF-κB pathway is strongly controlled by post-translational modification and subcellular compartmentalization as well as by interactions with other co-factors or co-repressors. For instance, the absence of Ripk1 in complex 1 of TNFR1 aligns with less nuclear translocation of NF-κB proteins and reduced expression of NF-κB target genes. Gene expression of NF-κB is auto-regulated as promoters of NF-κB subunit genes often contain NF-κB binding sites [49–54]. Alterations in the pathway may influence the expression of NF-κB genes in positive and negative ways. Here, NF-κB can act as an inducer or repressor depending on the nature of the dimer. In addition, other transcription factors, which can be differentially regulated in Ripk1-deficient Treg cells, participate in the regulation of NF-κB genes. For example, our scRNASeq data showed upregulation of the Ets family members Ets1 and Elf-1 in Ripk1-deficient Treg cells, which have been described as positive regulators of *Nfkb1* (p50) promoter activity [55]. Thus, the elevated expression of the *Nfkb1* positive regulators Ets1 and Elf-1 could contribute to the observed p50 upregulation in Ripk1-deficient Treg cells.

In our study, we show the impact of the YFP/Cre knock-in inserted in the *Foxp3* locus of the widely-used *Foxp3*^*Cre*^ mouse line. By using Ripk1-proficient chimeric mice, we observed that the eTreg subpopulation in the Cre-expressing Treg cells is reduced and that these cells display downregulation of classical Treg markers independently of Ripk1 expression. Although the deleterious effects of the *Foxp3*^*Cre*^ line have to be pondered, the sole presence of the YFP/Cre knock-in in the *Foxp3* locus cannot explain the phenotype that is observed by the deficiency of Ripk1 in Treg cells. For example, Ripk1-proficient Cre-homozygous female mice and Cre-hemizygous male mice do not develop any spontaneous autoimmune phenotype, in contrast to the *Ripk1*^Δ*Foxp3*^ mice. Moreover, the reduction of the eTreg population is more drastic in the Ripk1-deficient YFP^+^ Treg compartment of the *Ripk1*^*fl/fl*^ *Foxp3*^*Cre/wt*^ mice than in the Ripk1-proficient YFP^+^ Treg compartment of the *Ripk1*^*wt/wt*^ *Foxp3*^*Cre/wt*^ mice (Fig. 4G). In summary, our findings demonstrate that Ripk1 is essential to maintain the highly suppressive eTreg cell subset, which is crucial to preserve the immune homeostasis of the organism.

## Materials and methods

### Mice

Ripk^1tm1.1Pvdb^, B6.129(Cg)-Foxp3^tm4(YFP/icre)Ayr^/J, Foxp3^tm1(CD2/CD52)Shori^, and B6.129-Gt(ROSA)26Sor^tm1(cre/ERT2)Tyj^/J mice have been previously described [56–59]. *Ripk1*^Δ*Foxp3*^ mice and *Ripk1*^*fl/fl*^ *Foxp3*^*Cre/wt*^ mice were generated by crossing Ripk1^tm1.1Pvdb^ and B6.129(Cg)-Foxp3^tm4(YFP/icre)Ayr/^J. *Ripk1*^fl/fl^ Cre-ER^T2^ *Foxp3*^*hCD2*^ mice were generated by crossing Ripk1^tm1.1Pvdb^ and Foxp3^tm1(CD2/CD52)Shori^ and B6.129-Gt(ROSA)26Sor^tm1(cre/ERT2)Tyj^/J. Mice were kept under pathogen-free conditions in the animal facility of the Helmholtz Center for Infection Research and the Ruhr-University Bochum.

### Histology

Samples were fixed in formaldehyde and embedded in paraffin. Sections of 3 mm were stained with H&E and evaluated in a blinded manner as described [60]. Whole sections of bone marrow (sternum) from *Ripk1*^Δ*Foxp3*^ and Foxp3^Cre^ mice (*n* = 5, each group) were analyzed by a veterinary pathology expert in a semi-quantitative manner, assigning scores of 1 (reduced), 2 (normal) and 3 (augmented) for erythropoiesis and granulopoiesis.

### Cytokine determination

Serum cytokine levels were determined using LEGENDplex™ MU Th Cytokine Panel (12-plex) w/ FP V03 (741043, BioLegend) following manufacturer´s protocol and a CytoFLEX LX (Beckman-Coulter).

### Flow cytometry

Dead cells were excluded by LIVE/DEAD Fixable Blue Dead Cell Stain staining (#L23105, ThermoFisher Scientific) for 30 min at 4 °C in the dark. After washing in PBS, Fc receptors were blocked by 15 min incubation with TruStain FcX (#101320, BioLegend) in FACS buffer (2% BSA in PBS) at 4 °C. For surface staining, cells were resuspended in 100 µL FACS buffer containing the respective antibodies and incubated for 15 min at 4 °C in the dark. Cells were then washed with 500 µL FACS buffer and analyzed in a LSRII flow cytometer (BD Biosciences) or in a CytoFLEX LX (Beckman-Coulter). In case of analysis of intracellular proteins, cells were fixed, permeabilized and stained using Foxp3 Staining Buffer Set (#130-093-142, Miltenyi Biotec). The flow cytometry results were analyzed using FlowJo™ v10.8 Software (BD Life Sciences).

For viability analysis, after surface staining, cells were incubated with annexin-V (#640906, BioLegend) and 7-AAD (#420404, BioLegend) in the dark for 20 min. Active apoptosis was determined using CellEvent Caspase 3/7 Green (#C10427, ThermoFisher Scientific) or CellEvent Caspase 3/7 Red (#C10430, ThermoFisher Scientific). Antibodies (anti-mouse) used for flow cytometry: CD4-PacificBlue (RM4-5, #100531, BioLegend), CD8-BrilliantViolet605 (53-6.7, #100743, BioLegend), CD8-V500 (53-6.7, # 560776, BD Biosciences), CD19-PerCP/Cyanine5.5 (eBio1D3, #45-0193-82, eBiosciences), CD19-Alexa700 (eBio1D3, #67-0193-82, Invitrogen), NK1.1-PE (PK136, #553165, Becton, Dickinson), CD3-APC (145-2C11, #100312, BioLegend), CD3-PE-CF594(145-2C11, #562332, BD Biosciences), CD95-FITC (Jo, #554257, BD Biosciences), CD138-BrilliantViolet711 (281-2, #142519, BioLegend), CD185- BrilliantViolet605 (L138D7, #145513, BioLegend), CD279-ACP/Cy7 (29 F.1A12, #135224, BioLegend), GL7 (GL7, #144620, BioLegend), Foxp3-AlexaFluor488 (FJK-16s, #53-5773-82, eBiosciences), Foxp3-APC (REA788, #130-111-679, Miltenyi Biotech), Foxp3-PE (150D, #320007, BioLegend), anti-GFP-PE (FM264G, #338004, BioLegend), anti-GFP-AlexaFluor647 (FM264G, #338006, BioLegend), CD11b-PacificBlue (M1/70, #101224, BioLegend), F4/80-PE (BM8, #123110, BioLegend), F4/80-APC (BM8, #123116, BioLegend), Ly6G-PECy7 (1A8, # 127618, BioLegend), CD11c-APC/Cy7 (N418, # 47-0114-82, eBiosciences), CD62L-PerCP/Cyanine5.5 (MEL-14, # 45-0621-82, eBiosciences), CD62L-BrilliantViolet510 (MEL-14, #104441, BioLegend), CD44-APC (IM7, # 103012, BioLegend), CD44-PE/Cy7 (IM7, # 103030, BioLegend), CD44-APC/Cy7 (IM7, # 103027, BioLegend), SiglecF-PE (S17007L, #155506, BioLegend), TIGIT-PE/Cy7 (1G9, # 142108, BioLegend), ICOS-BrilliantViolet785 (C398.AA, #313534, BioLegend), CD103-Alexa700 (2E7, #121441, BioLegend), CD103-APC (2E7, #17-1031-82, eBiosciences), Ki67-Alexa700 (16A8, #652420, BioLegend), CTLA-4-PE (UC10-4B9, #106306, BioLegend), CD25-PE/Cy7 (PC61, #561780, Becton, Dickinson), CD25-APC (PC61, #102012, BioLegend), B220-PE (RA3-6B2, #553089, Becton, Dickinson), KLRG1-PE/Cy7 (2F1/KLRG1, #138416, BioLegend), Gpr15-PE/Dazzle594 (S15042I, #154609, BioLegend), Helios-PE-Dazzle594 (22F6, #137232, BioLegend) and RORγt-PE (Q31-378, #562607, BD Biosciences). Antibodies (anti-human) used for flow cytometry: CD2-PE (RPA-2.10, #300207, BioLegend).

### Western blot analysis

Cells were lysed by incubation in TPNE buffer (1x PBS, 300 mM NaCl, 1 mM EDTA, 1% Triton X-100) supplemented with protease inhibitors for 20 min on ice. After centrifugation, protein concentration was determined by BCA assay (ThermoFisher Scientific). Protein lysates were loaded onto 12% SDS-polyacrylamide gels, blotted onto PVDF membrane (GE Healthcare) and detected by chemiluminescence. Antibodies used for Western blotting: Ripk1 (3493S, Cell Signaling Technology), Beta-Actin (66009-1-lg, Proteintech), Alpha-Tubulin (66031-1-Ig, Proteintech), GAPDH (60004-1-Ig, Proteintech).

### Ex vivo cell sorting

Treg cells from *Ripk1*^fl/fl^ Cre-ER^T2^ *Foxp3*^*hCD2*^ mice and *Ripk1*^wt/wt^ Cre-ER^T2^ *Foxp3*^*hCD2*^ mice were purified using CD4^+^ CD25^+^ regulatory T cell isolation kit for magnetic separation (130-091-041; Miltenyi Biotec) according to manufacturer’s protocol. To increase Treg cell purity, anti-hCD2-PE (#300208, BioLegend) was used instead of the anti-CD25-PE provided in the kit. For RNA sequencing and pyrosequencing applications, CD4^+^ CD25^+^ Treg cells of *Ripk1*^*fl/fl*^ *Foxp3*^*Cre/wt*^ mice were sorted based on YFP expression using a FACSAria (BD Bioscience). For that purpose, single-cell suspensions from pooled spleen and lymph nodes of *Ripk1*^*fl/fl*^ *Foxp3*^*Cre/wt*^ mice were stained with anti-CD4-PacificBlue (#100531, BioLegend) and CD25-APC (#101910, BioLegend) in PBS for 15 min at 4 °C in the dark.

### Isolation of intestinal cells

Lamina propria immune cells and intraepithelial lymphocytes were isolated using Lamina Propria Dissociation Kit, mouse (130-097-410, Miltenyi Biotec) and gentleMACS Octo Dissociator with Heaters (130-096-427, Miltenyi Biotec) following manufacturer´s instructions.

### Cell culture and cell stimulation

Treg cells were seeded in RPMI 1640 medium (#11530586, Fisher Technologies) supplemented with 10% FBS (PAA Laboratories), 50 mM 2-mercaptoethanol, 50 mg/mL each of penicillin and streptomycin, 1% non-essential amino acids, and 1 mM sodium pyruvate (all from Life Technologies). For Treg cell activation, Treg Expansion Kit, mouse (130-095-925, Miltenyi) was used in the presence of 100 ng/ml murine IL-2 (402-ML, R&D). rmTNF (12343014, Immunotools) in a concentration of 50 ng/ml was used to stimulate Treg cells.

### Tamoxifen-induced Ripk1 deletion

To induce in vitro Ripk1 deletion in cells from *Ripk1*^fl/fl^ Cre-ER^T2^ *Foxp3*^*hCD2*^ mice, cells were seeded in supplemented RPMI 1640 medium (see cell culture section for details) and treated with 1 µM 4-Hydroxytamoxifen (H7904-5MG, Merck).

### Proliferation analysis

FACS sorted Treg cells were stained with cell trace violet (CTV, LifeTechnologies). Briefly, cells were washed two times with 1x PBS and re-suspended in 1 ml of 1x PBS. CTV was added to a final concentration of 5 μM and cells were incubated for 20 min at 37 °C in the dark. Subsequently, cells were stimulated with 2 μg/ml plate-bound anti-CD3 and 4 μg/ml soluble anti-CD28 plus 100 ng/ml IL-2. After 72 h cells were analyzed in a CytoFLEX LX flow cytometer (Beckman-Coulter).

### In vitro Treg cell induction from Ripk1-deficient naïve T cells

Naïve CD4^+^ T cells were isolated from pooled spleen and lymph node single-cell suspensions from *Ripk1*^fl/fl^ Cre-ER^T2^ *Foxp3*^*hCD2*^ and *Ripk1*^wt/wt^ Cre-ER^T2^ *Foxp3*^*hCD2*^ mice using Naive CD4^+^ T Cell Isolation kit (130-104-453, Miltenyi Biotec) according to manufacturer´s instructions. Subsequently, 1×10^6^ cells were seeded in a 24-well plate and treated with Tamoxifen (1 µM) to induce Ripk1 deletion or with the vehicle (ethanol). After 72 h, cells were washed and seeded in an anti-CD3 (#100238, BioLegend) pre-coated flat-bottom 96-well plate in a density of 1 × 10^5^ cells/well with iTreg polarizing medium in the presence or absence (negative control) of TGF-β (2.5 ng/ml, #100-21-10UG, PeproTech). iTreg polarizing medium is supplemented RPMI 1640 medium (see cell culture section for details) with 10 ng/ml murine IL-2 (402-ML, R&D), 10 ng/ml anti-IFNγ (self-made), 10 ng/ml anti-IL-4 (self-made), and 2 µg/ml anti-CD28 (#102116, BioLegend). After 5 days, cells were harvested and stained with LIVE/DEAD Fixable Blue Cell Stain (#L23105, ThermoFisher Scientific), anti-CD4-Pacific Blue (#100531, BioLegend), anti-hCD2-PE (#300208, BioLegend), and Foxp3-APC (130-111-679, Miltenyi Biotec) according to procedure described in flow cytometry analyses section. Treg cell differentiation was determined by measuring co-expression of Foxp3 and hCD2 using a CytoFLEX LX (Beckman-Coulter) flow cytometer.

### Single-cell RNA sequencing

The single-cell suspension was loaded onto a well on a 10x Chromium Single Cell instrument (10x Genomics). Barcoding and cDNA synthesis were performed according to the manufacturer’s instructions. Briefly, the 10x GemCode Technology partitions thousands of cells into nanoliter-scale Gel Bead-In-EMulsions (GEMs). GEMs are generated by combining barcoded Single Cell 3’ Gel Beads (v3.1), a Master Mix containing CellPlex labeled cells, and Partitioning Oil onto Next GEM Chromium Chip G. The poly(dT) and the Capture Sequence 2 primers on the gel bead are engaged simultaneously in two different reactions inside individual GEMs, generating barcoded, full-length cDNA from poly-adenylated mRNA and barcoded DNA from the CMO Feature Barcode. The 10x Barcoded cDNA molecules are then amplified via PCR, using compatible primers to generate sufficient mass for library construction. Size selection is used to separate the amplified cDNA molecules for 3’ Gene Expression and Cell Multiplexing library construction. Generated dual index cDNA libraries were sequenced on Illumina NovaSeq6000 with 10X Genomics dual indexing metrics: 28/10/10/90 for Read1/i7/i5/Read2. The sequencing depth was 25.000 reads per cell for 3’ Gene Expression and 5000 reads per cell for Cell Multiplexing library. Raw sequencing data were demultiplexed and further converted into a single-cell level gene counts matrix with Cell Ranger 7.0.0 multi with default parameters. Sequences were mapped to mm10-3.0.0 transcriptome (10X Genomics).

### Bioinformatic analysis of single-cell RNA sequencing

The single-cell data was further processed with the Seurat_5.0.1 pipeline in Rstudio version 4.3.3. Cells expressing CD19 were excluded from the RNA data for downstream analysis. Expression data was normalized and scaled with SCTransform applying the default 3000 nfeature parameter. During normalization, confounding sources of variation (mitochondrial mapping percentage) were removed. The cell type identity of the clusters was annotated with the SingleR_2.4.1 package. As a reference, the mouse bulk expression data from the Immunologic Genome Project (ImmGenData) was used. For differential Expression the Seurat function FindMarkers was used. AverageExpression was used to get the average scaled counts of the genes across the different cells in the Treg clusters.

### DNA methylation analysis

Genomic DNA was isolated from sorted Treg cells using the DNeasy Blood & Tissue Kit (Qiagen) and converted by bisulfite by using the EZ DNA Methylation-Lightning Kit (Zymo Research) according to the manufacturer’s protocol. The TSDR/CNS2 was amplified by PCR using bisulfite-converted DNA, the primers mTSDR-for (5′-bio-TAAGGGGGTTTTAATATTTATGAGGTTT-3′) and mTSDR-rev (5′-CTAAACTAACCAACCAACTTCCTA-3′) and the ZymoTaq PreMix (Zymo Research) following the manufacturer’s protocol. The amplification product was sequenced by pyrosequencing, using the sequencing primer mTSDR-seq0 (5′- CCATACAAAACCCAAATTC -3′), mTSDR-seq1 (5′-ACCCAAATAAAATAATATAAATACT-3′), mTSDR-seq2 (5′-ATCTACCCCACAAATTT-3′), or mTSDR-seq3 (5′-AACCAAATTTTTCTACCATT-3′) on a Pyromark Q24 (Qiagen, Hilden, Germany) and analyzed following the manufacturer’s instructions. Characterized CpG motifs are located on chromosome X:7450102-7450559 (Foxp3) (GRCm39).

### Statistical analysis

If not stated differently, statistical significance was calculated by two-sided Mann–Whitney or Kruskal–Wallis one-way ANOVA tests using GraphPad Prism v 10.4.1 (GraphPad Software, La Jolla, CA). *P* values considered significant as follows: *p < 0.05; **p < 0.01; ***p < 0.001, and ****p < 0.0001. When no normal distribution could be assumed, non-parametric tests such as Mann–Whitney U test were performed. No estimation of variance was done. Size of animal groups were calculated using the G*Power software for statistical power analysis provided by the University of Düsseldorf, Germany. Animals were allocated to the experimental groups based on the genotype. No randomization for animal allocation was performed. Mice with unwanted genotype were generated due to the breeding strategy. Mice with unwanted genotype were excluded from the study. For biochemical, flow cytometry or molecular biological analyses of mouse samples, no blinding was done.

## Supplementary information

Supplemental material

Original Data Files

## Data Availability

RNA-Seq and scRNA-Seq data were deposited to the Gene Expression Omnibus repository under the accession number GSE271416.
