# Supplementary material for: Ripk1 is critical for preserving effector regulatory T cells and the suppressive transcriptional program in regulatory T cells
Source: Cell Death Differ. 2025 Jul 22;33(2):284–97. doi: 10.1038/s41418-025-01550-3 (PMC12881586; doi:10.1038/s41418-025-01550-3)
Supplement: Supplementary file 1 — Supplemental material [file 41418_2025_1550_MOESM1_ESM.pdf]

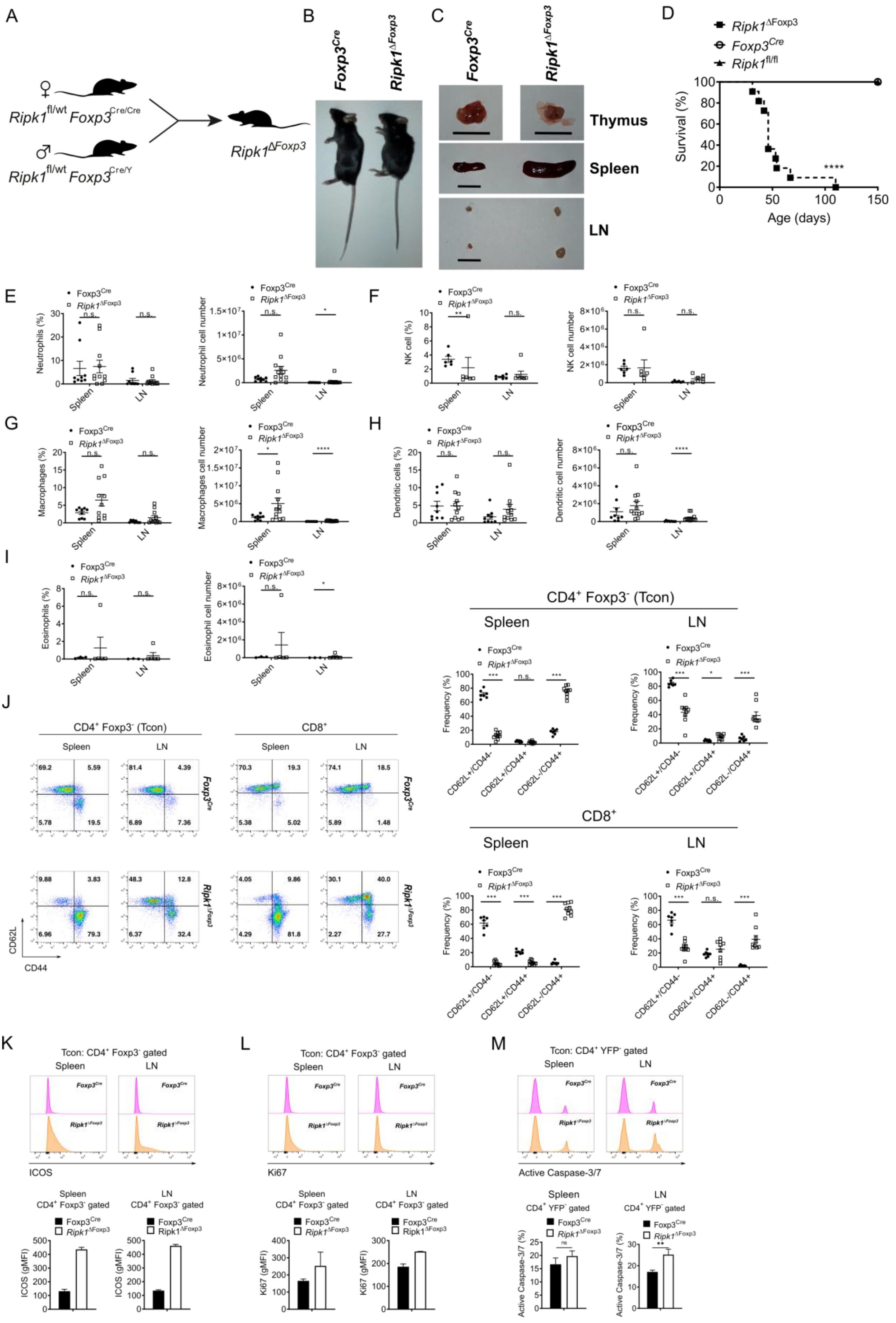

Figure S1. (A) Schematic representation of the generation of the Foxp3-specific knock-out mouse *Ripk1*$^{\Delta Foxp3}$ from *Ripk1*$^{fl/fl}$ and *Foxp3*^{Cre} mice. Y next to Cre stands for Y chromosome representing hemizygous male mice. (B) Picture of 9-week-old *Ripk1*$^{\Delta Foxp3}$ and *Foxp3*^{Cre} mice. (C) Pictures of thymus, spleen and lymph nodes of 9-week-old *Ripk1*$^{\Delta Foxp3}$ and *Foxp3*^{Cre} mice. Scale bar = 1 cm. (D) Survival curve of *Ripk1*$^{\Delta Foxp3}$ (n=11), *Foxp3*^{Cre} (control, n=6) and *Ripk1*$^{fl/fl}$ (control, n=6) mice. The

defined humane endpoint was counted as “death” of the animals. Statistical analyses were performed by Log-rank (Mantel-Cox) test. ****p < 0.0001. Percentages (left) and cell number (right) of neutrophils (E), NK cells (F), macrophages (G), dendritic cells (H) and eosinophils (I) of spleen and lymph nodes (LN) from *Ripk1*$^{\Delta Foxp3}$ and *Foxp3*^{Cre} mice. (E-I) Mean ± SEM are given. Each symbol represents a single mouse in the scatter plots. (J) Representative dot plots (left) and frequencies (right) of T cell activation markers CD62L and CD44 in CD4^{+} Foxp3^{-} and CD8^{+} cells from spleen and lymph nodes (LN) of *Ripk1*$^{\Delta Foxp3}$ and *Foxp3*^{Cre} mice. Representative histograms and geometric mean fluorescence intensity (gMFI) bar graphs of ICOS (K) and Ki67 (L) expression in CD4^{+} Foxp3^{-} cells from spleen and lymph nodes (LN) of *Ripk1*$^{\Delta Foxp3}$ and *Foxp3*^{Cre} mice (n=2, each). (M) Representative histograms and summary bar graph of active caspase-3/7 in CD4^{+} Foxp3^{-} cells from spleen and lymph nodes (LN) of *Ripk1*$^{\Delta Foxp3}$ and *Foxp3*^{Cre} mice (n=5, each). (E-I, M) Statistical analyses were performed by two-tailed Mann-Whitney tests. *p < 0.05, **p < 0.01, ****p <0.0001, n.s. = not significant.

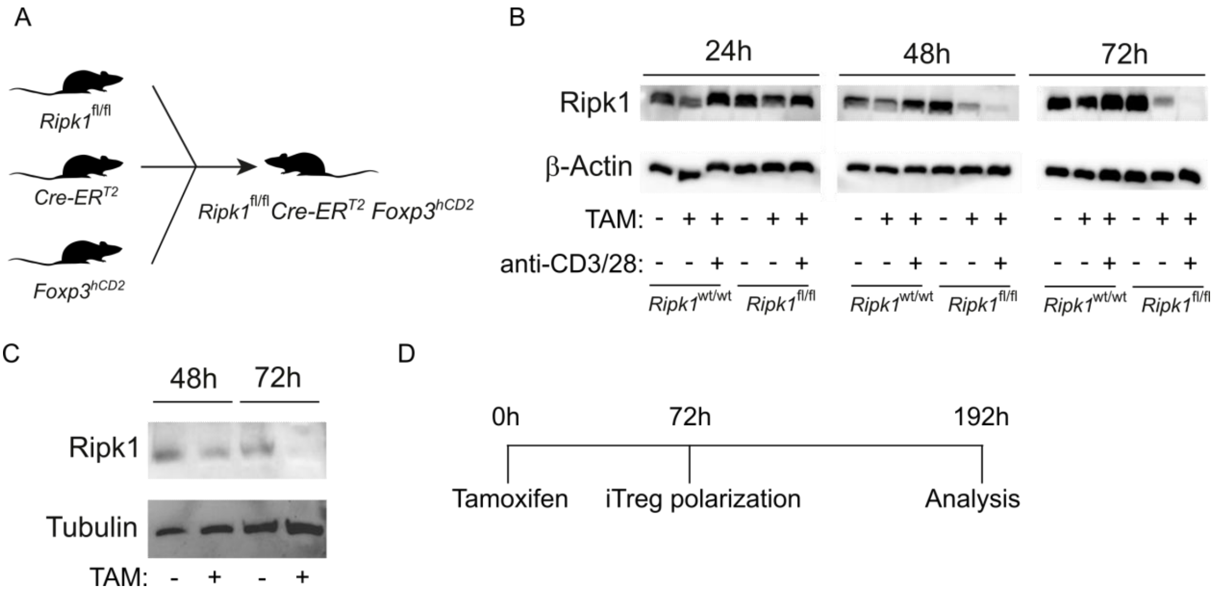

Figure S2. (A) Schematic representation of the generation of Ripk1 inducible murine cells from *Ripk1*$^{fl/fl}$ Cre-ER^{T2} *Foxp3*^{hCD2} mice using *Ripk1*$^{fl/fl}$, Cre-ER^{T2} and *Foxp3*^{hCD2} mice. (B) Immunoblot analysis of Ripk1 in lymphocytes isolated from lymph nodes of *Ripk1*$^{fl/fl}$ Cre-ER^{T2} *Foxp3*^{hCD2} (*Ripk1*$^{fl/fl}$) and *Ripk1*$^{wt/wt}$ Cre-ER^{T2} *Foxp3*^{hCD2} (*Ripk1*$^{wt/wt}$ control) mice in the indicated time points. Cells were treated with tamoxifen or vehicle and were stimulated with anti-CD3 and anti-CD28 beads or left unstimulated. (C) Immunoblot analysis of Ripk1 in purified Treg cells from *Ripk1*$^{fl/fl}$ Cre-ER^{T2} *Foxp3*^{hCD2} mice stimulated with anti-CD3 and anti-CD28 and treated with tamoxifen or the vehicle for the indicated time points. (D) Scheme of *in vitro* iTreg polarization assay after tamoxifen-induced deletion of Ripk1 in naïve Tcon cells (Figure 3C).

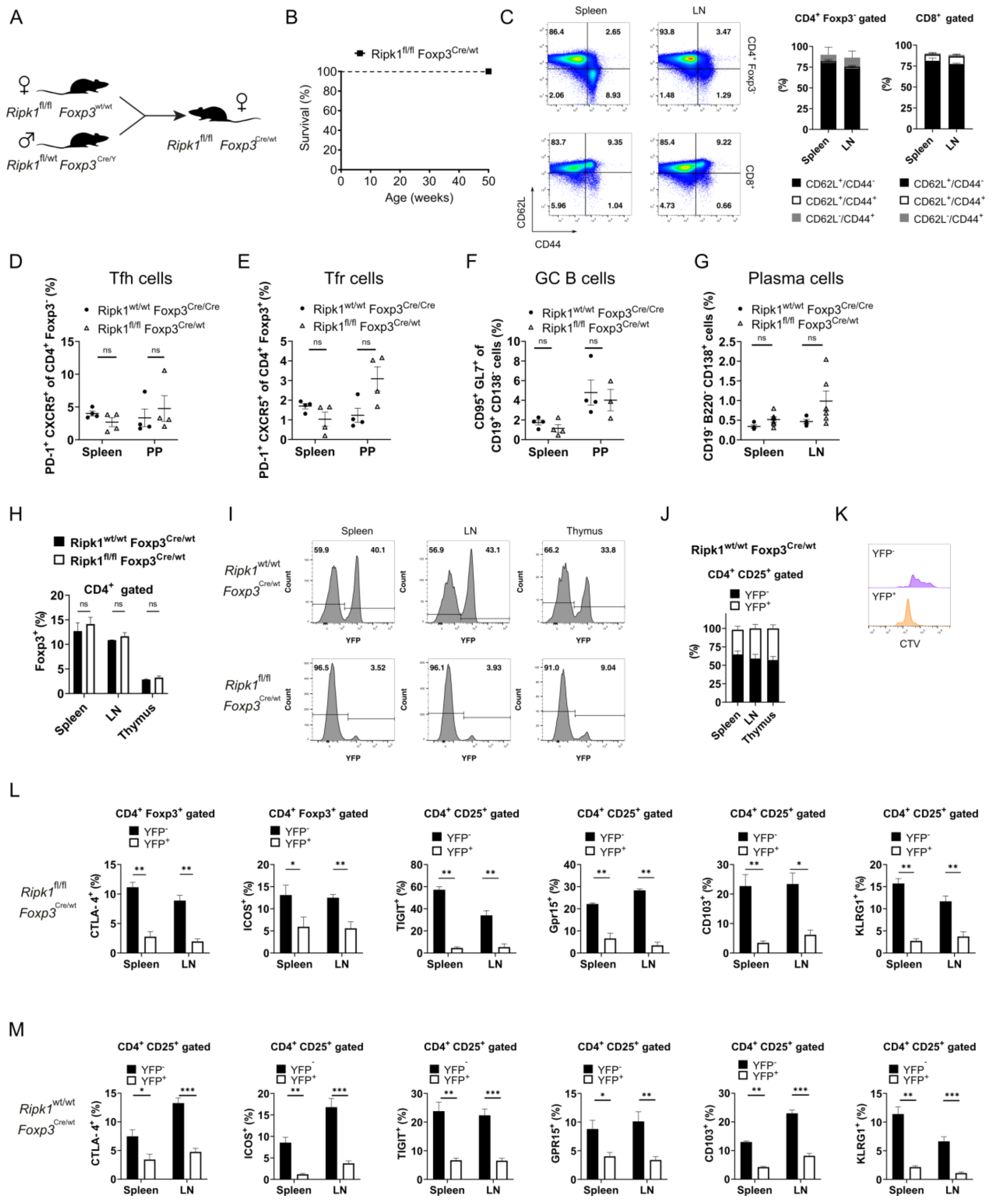

Figure S3. (A) Schematic representation of the generation of female *Ripk1*$^{fl/fl}$ *Foxp3*$^{Cre/wt}$ chimeric mice. (B) Survival curve of Ripk1$^{fl/fl}$ Foxp3$^{Cre/wt}$ mice (n=9). (C) Representative dot plots (left) and frequencies (right) of T cell activation markers CD62L and CD44 in CD4^{+} Foxp3^{-} and CD8^{+} cells from spleen and lymph nodes (LN) of *Ripk1*$^{fl/fl}$ *Foxp3*$^{Cre/wt}$ mice (n=3). Frequencies of T follicular helper (Tfh) cells (D), T follicular regulatory (Tfr) cells (E) and germinal center (GC) B cells (F) in spleen and Peyer´s patches (PP) of *Ripk1*$^{fl/fl}$ *Foxp3*$^{Cre/wt}$ and *Ripk1*$^{wt/wt}$ *Foxp3*$^{Cre/Cre}$ female mice. (G) Frequencies of plasma cells in spleen and lymph nodes of *Ripk1*$^{fl/fl}$ *Foxp3*$^{Cre/wt}$ and *Ripk1*$^{wt/wt}$ *Foxp3*$^{Cre/Cre}$ female mice. (D-G) Each symbol represents a single mouse in the scatter plots. (H) Frequency of Foxp3^{+} cells within CD4^{+} cells in thymus, spleen and lymph nodes (LN) of *Ripk1*$^{wt/wt}$ *Foxp3*$^{Cre/wt}$ mice (n=3) and *Ripk1*$^{fl/fl}$ *Foxp3*$^{Cre/wt}$ mice (n=11). (I) Representative histograms of YFP^{-} and YFP^{+} cell distribution in *Ripk1*$^{wt/wt}$ *Foxp3*$^{Cre/wt}$ mice and *Ripk1*$^{fl/fl}$ *Foxp3*$^{Cre/wt}$ mice. (J) Summary bar graph of the distribution of percentages of YFP^{-} and YFP^{+} cells within the CD4^{+} Foxp3^{+} population of *Ripk1*$^{wt/wt}$ *Foxp3*$^{Cre/wt}$ mice (n=3). (K) Histograms of CTV dilution from FACS sorted CD4^{+} CD25^{+} YFP^{-} and CD4^{+} CD25^{+} YFP^{+}

cells of *Ripk1*$^{fl/fl}$ *Foxp3*$^{Cre/wt}$ mice (n=4) stimulated with anti-CD3 (1 µg/ml), anti-CD28 (2 µg/ml) and IL-2 (10 ng/ml) for 72 h. Summary bar graphs of the indicated markers (CTLA-4, ICOS, TIGIT, Gpr15, CD103 and KLRG1) in YFP$^-$ and YFP$^+$ cells within CD4$^+$ CD25$^+$ cells from spleen and lymph nodes (LN) of (L) *Ripk1*$^{fl/fl}$ *Foxp3*$^{Cre/wt}$ mice (n=5) and (M) *Ripk1*$^{wt/wt}$ *Foxp3*$^{Cre/wt}$ mice (n=7). Statistical analyses were performed by two-tailed Mann-Whitney tests. *p < 0.05, **p < 0.01, ***p <0.001, n.s. = not significant.

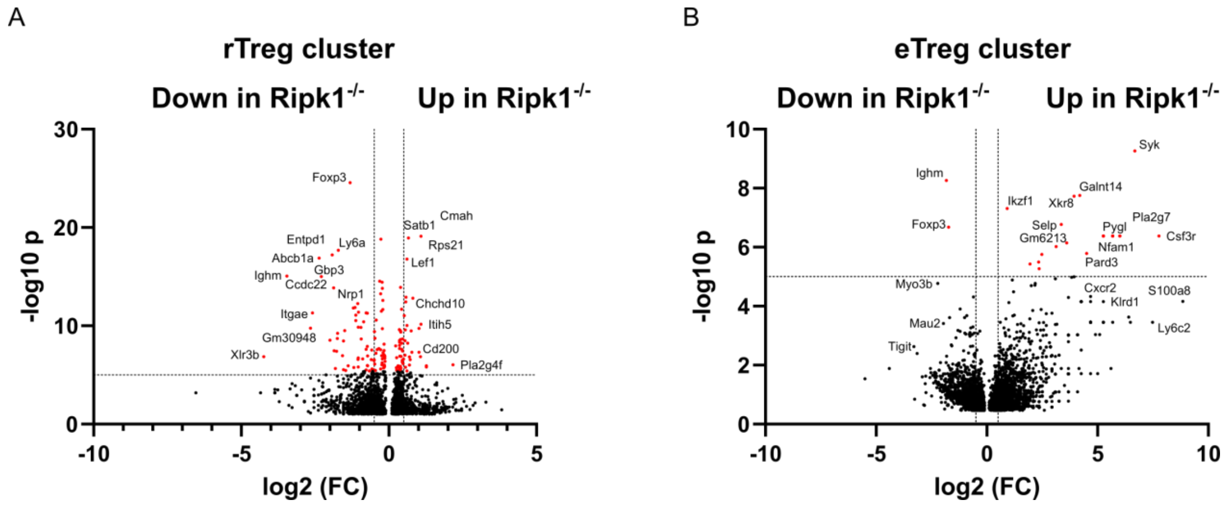

Figure S4. (A) scRNASeq volcano plot showing changes in gene expression of rTreg cells (Red labeled in the Seurat clustering of Figure 5) of CD4$^+$ CD25^{hi} YFP$^+$ (Treg Ripk1$^{-/-}$) cells vs. CD4$^+$ CD25^{hi} YFP$^-$ (Treg Ripk1$^{+/+}$) cells. (B) scRNASeq volcano plot showing changes in gene expression of eTreg cells (Green labeled in the Seurat clustering of Figure 5) of CD4$^+$ CD25^{hi} YFP$^+$ (Treg Ripk1$^{-/-}$) cells vs. CD4$^+$ CD25^{hi} YFP$^-$ (Treg Ripk1$^{+/+}$) cells.
