# Supplementary material for: Ripk1 is critical for preserving effector regulatory T cells and the suppressive transcriptional program in regulatory T cells
Source: Cell Death Differ. 2025 Jul 22;33(2):284–97. doi: 10.1038/s41418-025-01550-3 (PMC12881586; doi:10.1038/s41418-025-01550-3)
Supplement: Supplementary file 2 — Original Data Files [file 41418_2025_1550_MOESM2_ESM.pdf]

Original data

Original western blots

Figure 1A

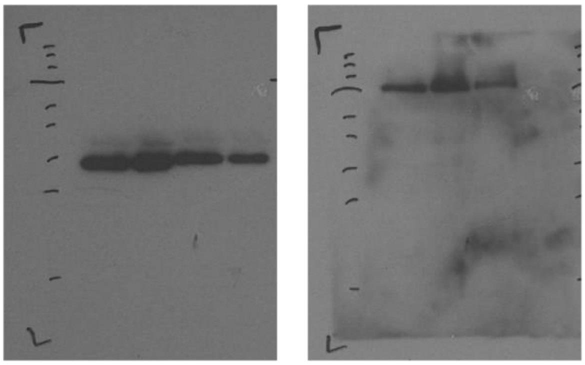

GAPDH Ripk1

Figure 3A

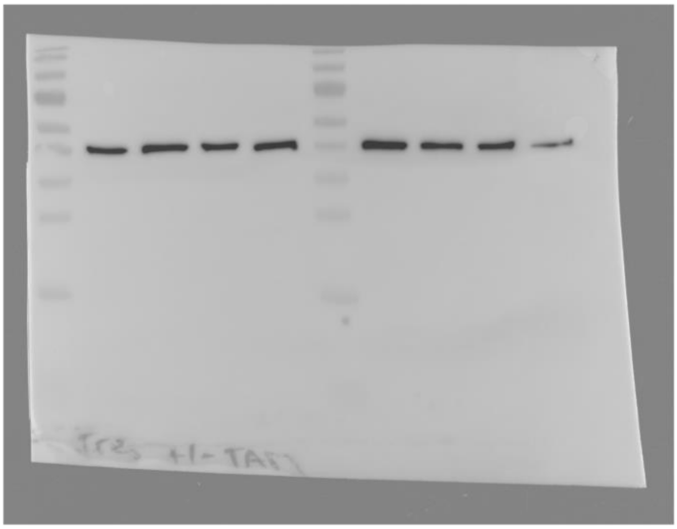

β-Actin

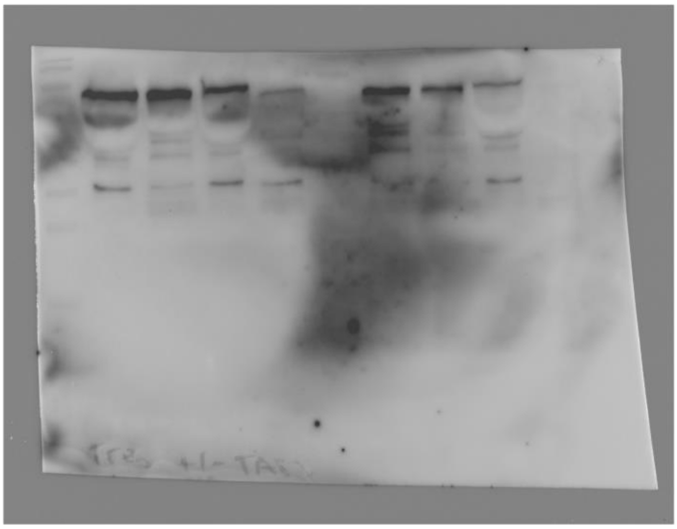

Ripk1

Figure S2B

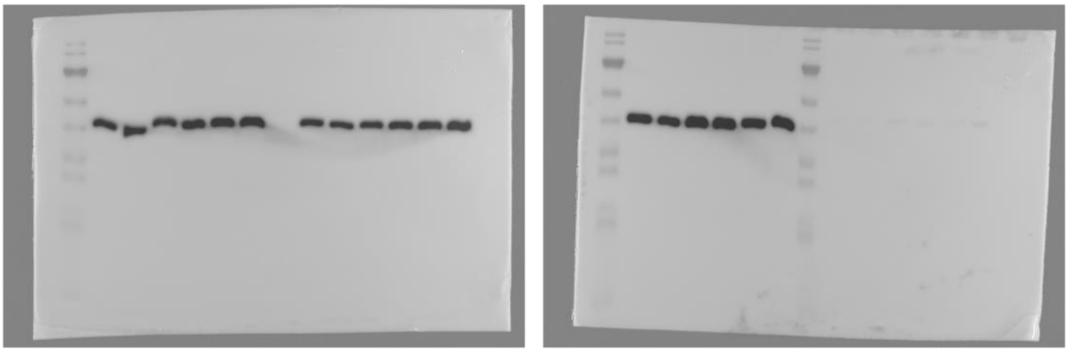

β-Actin

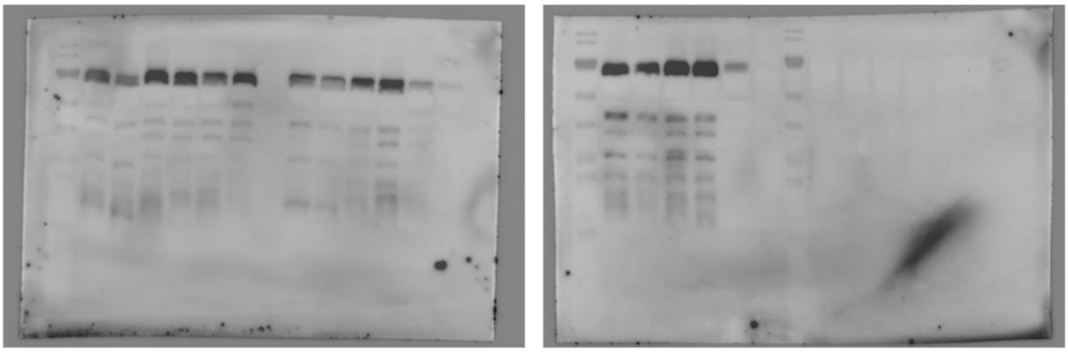

Ripk1

Figure S2C

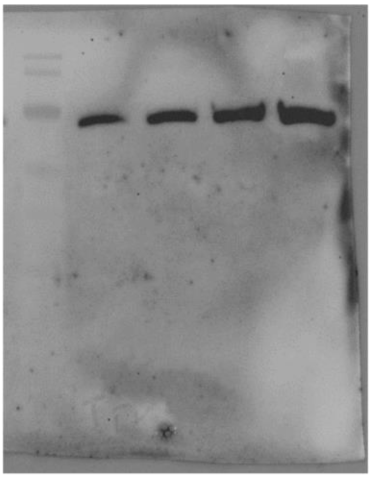

Tubulin

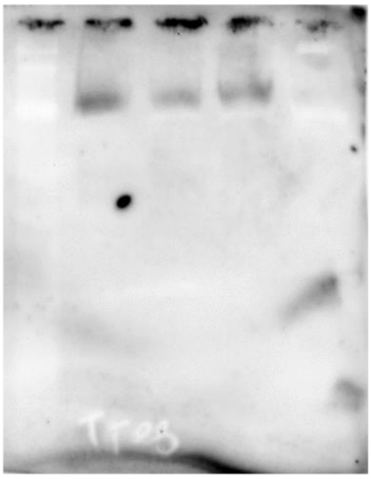

Ripk1
